# A Pendulum-like Low Frequency Electromagnetic Vibration Energy Harvester Based on Polymer Spring and Coils

**DOI:** 10.3390/polym13193380

**Published:** 2021-09-30

**Authors:** Yunjia Li, Xinyi Wang, Shuhan Zhang, Chenyuan Zhou, Dayong Qiao, Kai Tao

**Affiliations:** 1 School of Electrical Engineering, Xi’an Jiaotong University, Xi’an 710049, China; xywang@stu.xjtu.edu.cn (X.W.); zhangshuhan123@foxmail.com (S.Z.); zhouchenyuan@stu.xjtu.edu.cn (C.Z.); 2 Micro and Nano Electromechanical Systems Laboratory, Northwestern Polytechnical University, Xi’an 710072, China; dyqiao@nwpu.edu.cn

**Keywords:** polymer beams, vibration sensors, vibration energy harvester

## Abstract

This paper presents a low-frequency electromagnetic vibrational energy harvester (EVEH) with two degrees of freedom and two resonant modes. The proposed EVEH is based on a disc magnet suspended in a pendulum fashion by a polymeric spring between two sets of polymer coil stacks. The fabricated EVEH is capable of harvesting vibration energy on two directions with an extended bandwidth. With a sinusoidal acceleration of ±1 g on Z direction, a peak-to-peak closed-circuit output voltage of 0.51 V (open-circuit voltage: 1 V), and an output power of 35.1 μW are achieved at the resonant frequency of 16 Hz. With a sinusoidal acceleration of ±1.5 g on X direction, a peak-to-peak output voltage of 0.14 V and power of 2.56 μW are achieved, at the resonant frequency of 20 Hz.

## 1. Introduction

Electromagnetic vibrational energy harvesters (EVEHs) are devices that convert ambient vibrations to electrical power, often by the relative movement between a permanent magnet and a solenoid coil [1,2,3,4]. Compared with its piezoelectric and electrostatic counterparts, the EVEHs possess advantages such as simpler design and structure, higher output power, and easier fabrication [5]. The EVEHs can be implemented by using macroscopic components, printed circuit board (PCB) technology [6,7,8,9], and microelectromechanical systems (MEMS) technologies [10,11,12].

The primary goals for designing the EVEHs are high output power, small volume, and low cost. However, in order to be used in practical applications, it is essential to match the resonant frequency of the EVEHs to the ambient vibration. It is known that the ambient vibrations where EVEHs can be used are mostly located in the frequency range of 1–200 Hz [11,12,13,14]. Therefore, it is desirable to implement EVEHs with low resonant frequencies. However, the stiffness of springs scales inversely with the geometrical dimensions, hence it is often difficult to reduce the resonant frequency (in direct proportional to the square root of the spring stiffness) of the miniaturized EVEHs. The conventional strategies to reduce the resonant frequency is to use low-stiffness material as springs or use folded spring design [11,14], which usually results in EVEHs with resonant frequency above 100 Hz. EVEHs with resonant frequency lower than 100 Hz is less reported [15].

Besides the low frequency nature of ambient vibrations, many of them are multi-directional, such as the movement of human, vehicles (ships and airplanes), and cables. In addition, many of the vibrations vibrates on the horizontal direction with respect to the ground, such as power transformers. However, most of the reported EVEHs are based on unidirectional operation solely on the vertical direction. Therefore, it is desirable to obtain EVEHs that can harvest energy from vibrations of different directions, especially on the horizontal direction. Nevertheless, energy harvesters capable of harvesting vibrations on different directions are not so often reported. The reported multi-directional devices are mainly piezoelectric energy harvesters based on a cantilever structure [16,17,18]. EVEHs for multi-directional vibrations have rarely been reported, according to the best knowledge of the authors.

It is intuitive and straightforward to design and implement a multi-directional VEH by using a cantilever structure because its free end can move in any direction. Nevertheless, piezoelectric cantilevers cannot be implemented with large proof mass in order to avoid cracks and initial deflection, which limits the lower bound of its resonant frequency. In this work, we adopt the single-end structural feature of the cantilevers, but change the operation direction: suspending a permanent magnet in a pendulum-like fashion by a single flexible polymeric spring between two sets of stacked polymer coils. Compared to traditional EVEHs based on cantilever structures, the polymer single-string spring structure enables the integration of large proof mass, low operation frequency, and robustness of the device without the concern of crack or initial deflection. The stacked polymer coils enable integration of high-density coils within a small distance in the vicinity of the magnet, inducing large output voltage at low acceleration levels. The proposed EVEH is capable of generating rectifiable output voltage at very low frequency on two directions with two resonant modes, which provides wider applicability. Moreover, the proposed structure is shown to operate reliably at high acceleration levels. The design, fabrication, and characterization of the EVEH device will be presented and discussed.

## 2. System Design

### 2.1. Concept of the EVEH

**Figure 1:**
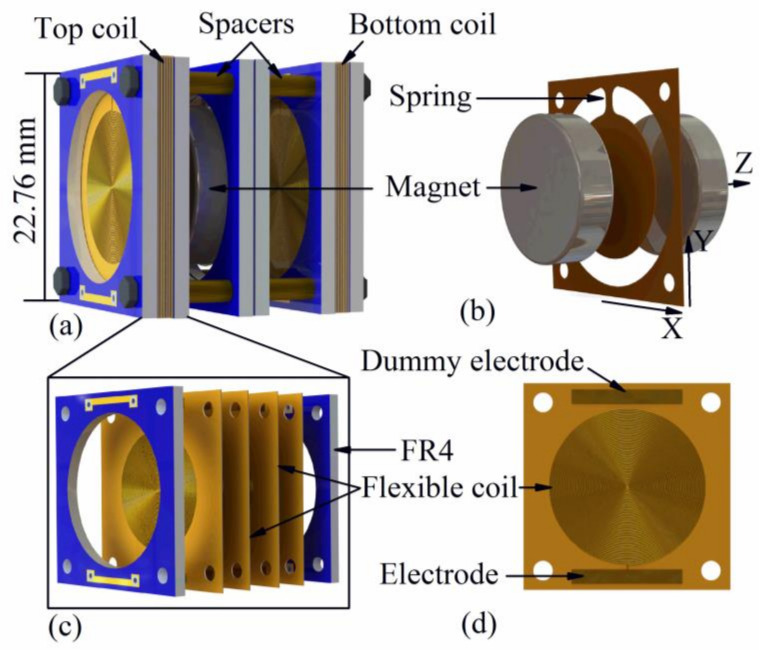
Schematic illustration of the: (**a**) EVEH device; (**b**) pendulum-like suspended magnet by polymer spring; (**c**) stacked polymer coils; and (**d**) a layer of polymer coils.

The combined usage of the pendulum structure, the polymer-based springs and stacked coils enables excellent performance and robustness of the proposed EVEH. A schematic illustration of the proposed EVEH device is shown in Figure 1. It is composed of disc permanent magnets suspended like a pendulum by a soft polyimide spring, between two sets of high-density polymer coil stacks. The magnet suspended between two coil stacks is composed of two identical disc N52 NdFeB magnets bound to each other by magnetic force. A thin circular polyimide layer connected to the spring is sandwiched between the two magnets. The spring is anchored to the polyimide frame clamped between two rigid FR4 frames. Each of the polymer coil stacks consists of 14 identical polymer planar coil layers connected in series, forming a high-density 3D coil stack [7]. Fourteen is the maximum number of layers that can be included in the device without open-circuit fault. The advantage of the coil stacking technology is low cost, batch fabrication, and easy assembly. The magnet layer and the coil stacks are assembled by using bolts and spacers. When the EVEH is subjected to external accelerations, the magnet oscillates and generates a changing magnetic field in the coils, which induces voltage in the coils. When the acceleration on X direction is applied, the magnet oscillates in a torsional mode (torsion of the spring). When the acceleration on Z direction is applied, the magnet oscillates in a bending mode (bending of the spring). The proposed EVEH is not sensitive to the vibration on Y direction, because the Y-direction movement of the magnet involves axial elongation of the spring, which requires a large load to take place.

The polyimide spring has very low stiffness compared to the cantilever-based energy harvesters. However, due to the flexible nature of polyimide, the spring is rather robust compared to many of the cantilever structures made of brittle materials (e.g., silicon and piezoelectric ceramics). Therefore, the polyimide spring is capable of supporting large movement of the magnet. When excited by Z-direction vibration, the magnet will bend the spring and oscillate in Z direction. At high acceleration levels, collisions between the magnet and the coil stack may occur in each oscillation cycle. This is different from most of the oscillating structures used for energy harvesting. The collisions between the magnet and the coil stack can be avoided by increasing the coil-magnet distance. However, the energy harvesting efficiency of the EVEH is significantly reduced when the coil-magnet distance increases, as a result of the exponentially decaying magnetic field outside the permanent magnet. In one of our previous study on the effect of the coil-magnet distance, the output voltage of reduces from 1.57 V at the coil-magnet distance of 0.71 mm [7] to around 0.5 V at 4 mm. This is a deteriorative effect especially for the EVEH working under low acceleration levels, which is the case for most of the ambient vibrations. Therefore, the designed coil-magnet distance is 2.5 mm, which is an intermediate value between 0.7 mm that we often use and 4 mm which is almost the lowest limit for the coil to harvest energy from the magnet. The value of 2.5 mm maintains a medium energy harvesting efficiency at a moderate collision level. The collision between the magnet and the coil may bring reliability issues after a long period of operation, and will be investigated in the future.

### 2.2. System-Level Model

Under the assumptions that the spring material is linearly elastic and that the shear deformation and rotary inertia are negligible, the differential equation of motion for a spring under axial load is given by [19]:

(1) $$EI\frac{\partial^{4}y}{\partial x^{4}}+F\frac{\partial^{2}y}{\partial x^{2}}+\rho A\frac{\partial^{2}y}{\partial t^{2}}=0$$

where *y* = *y*(*x*, *t*) is the transverse displacement of the spring located at the distance x from the left-hand end, *F* is the axial load. *EI* is the flexural rigidity of spring, *A* is the cross-sectional area, and *ρ* is the mass density of the spring material. If *F* = 0, Equation (1) reduces to the classical solution.

When the spring performs harmonic oscillations *y*(*x*, *t*) = *Y*(*x*)∙cos(*ωt*) the above expression becomes:

(2) $$EI\frac{d^{4}Y\left(x\right)}{dx^{4}}+F\frac{d^{2}Y\left(x\right)}{dx^{2}}-\rho A\omega^{2}Y\left(x\right)=0$$

where ω is the circular natural frequency. By introducing the dimensionless spring co-ordinate $\bar{x}=x/l$, the solution of Equation (2) is:

(3) $$\bar{Y}(\bar{x})=A\cosh(\alpha_{1}\bar{x})+B\sinh(\alpha_{1}\bar{x})+C\cos(\alpha_{2}\bar{x})+D\sin(\alpha_{2}\bar{x})$$

where *A*, *B*, *C*, and *D* are constant coefficients that can be obtained from certain boundary conditions, and α_1_ and α_2_ are defined as:

(4) $$\begin{array}{l} \alpha_{1}=l\sqrt{-\frac{F}{2EI}+\sqrt{{\left(\frac{F}{2EI}\right)}^{2}+\left(\frac{\rho A}{EI}\right)\omega^{2}}}=\sqrt{-\frac{{\bar{k}}^{2}}{2}+\sqrt{\frac{{\bar{k}}^{4}}{4}+{\bar{\beta }}^{4}}}\\ \alpha_{2}=l\sqrt{\frac{F}{2EI}+\sqrt{{\left(\frac{F}{2EI}\right)}^{2}+\left(\frac{\rho A}{EI}\right)\omega^{2}}}=\sqrt{\frac{{\bar{k}}^{2}}{2}+\sqrt{\frac{{\bar{k}}^{4}}{4}+{\bar{\beta }}^{4}}} \end{array}$$

where ${\bar{k}}^{2}=Fl^{2}/EI$ and ${\bar{\beta }}^{2}=\omega l^{2}\sqrt{\rho A/EI}$ are dimensionless factors.

The spring of EVEH loaded with heavy proof mass in this work can be modeled as a clamped-free beam with tensile axial load. The boundary conditions of the spring are given as follows:

(5) $$\mathrm{Clamped}\;\mathrm{end:}\;\begin{array}{cc} \bar{Y}\left(0\right)=0 & {\bar{Y}}^{\prime }\left(0\right)=0 \end{array}$$

(6) $$\mathrm{Free}\;\mathrm{end:}\;\begin{array}{cc} {\bar{Y}}^{\prime \prime }\left(l\right)=0 & {\bar{Y}}^{\prime \prime \prime }\left(l\right)+{\bar{k}}^{2}{\bar{Y}}^{\prime }\left(l\right)=0 \end{array}$$

Insert conditions (5) and (6) into Equation (3) yields the characteristic equation below:

(7) $$2{\bar{\beta }}^{4}+\left(2{\bar{\beta }}^{4}+{\bar{k}}^{4}\right)\cosh\alpha_{1}\cos\alpha_{2}-{\bar{\beta }}^{2}{\bar{k}}^{2}\sinh\alpha_{1}\sin\alpha_{2}=0$$

As *F* (or $\bar{k}$) increase, the frequencies decrease until *F* equals the critical or Euler’s buckling load *F_cr_* of the beam. The value of *F_cr_* can be obtained by putting ${\bar{\beta }}^{2}=0$ in Equation (7) which results in the relationship $\cos{\bar{k}}_{n}=0$. For the first mode:

(8) $$F_{cr}=\pi^{2}EI/4l^{2}$$

The Rayleigh expression for the fundamental natural frequency of unloaded uniform beam is:

(9) $$\omega_{0}^{2}=\int_{0}^{l}EI{\left(\frac{d^{2}y}{dx^{2}}\right)}^{2}dx/\int_{0}^{l}\rho Ay^{2}dx$$

The fundamental natural frequency of tensile beam is:

(10) $$\omega^{2}=\left(\int_{0}^{l}EI{\left(\frac{d^{2}y}{dx^{2}}\right)}^{2}dx+F\int_{0}^{l}{\left(\frac{dy}{dx}\right)}^{2}dx\right)/\int_{0}^{l}\rho Ay^{2}dx$$

The critical or Euler’s buckling load *F_cr_* for the same beam is:

(11) $$\int_{0}^{l}EI{\left(\frac{d^{2}y}{dx^{2}}\right)}^{2}dx+F\int_{0}^{l}{\left(\frac{dy}{dx}\right)}^{2}dx=0$$

Substituting Equations (9) and (11) into Equation (10) gives:

(12) $$f=f_{0}\sqrt{1+\frac{F}{F_{cr}}}$$

where *f*_0_ is the natural frequency of uncompressed beam, *f* is the fundamental natural frequency of tensile beam, *F_cr_* is the critical or Euler’s buckling load for beam, and *F* is the applied axial tensile load.

**Figure 2:**
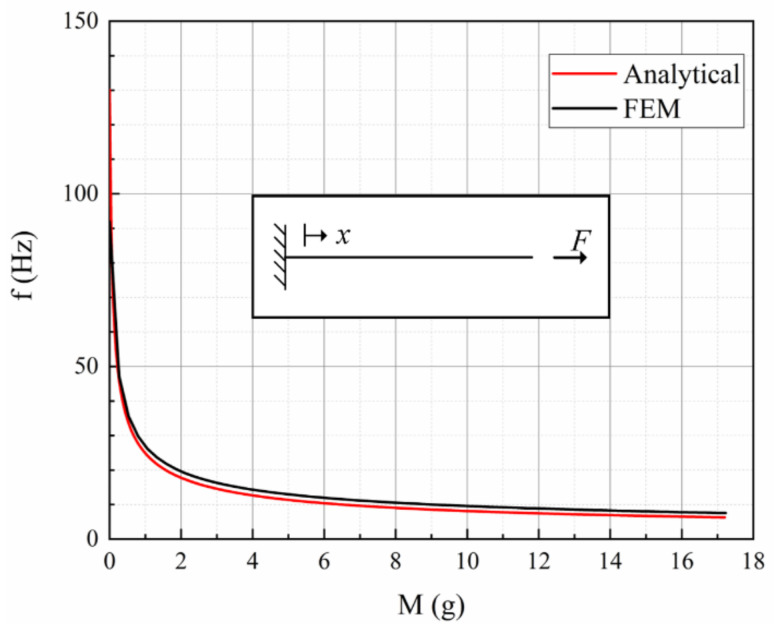
The fundamental resonant frequency versus the mass of the magnet. The computation is based on the values given in Table 1.

As the magnet asserts a large axial force to the spring, we assume the spring to be a clamped-free beam with uniform cross-section and constant axial tensile load. The factor that has been omitted in the model is the dynamic deformation of the spring during oscillation and the combined torsional movement of the spring. Therefore, the fundamental resonant frequency can be approximated is given by:

(13) $$f=\frac{1}{2\pi }\sqrt{\frac{k_{eff}\left(F_{cr}+F\right)}{\left(m+M\right)F_{cr}}}$$

where *k_eff_* is the effective stiffness of the spring calculated by the finite-element method (FEM), *m* is the total mass of the beam, and *M* is the mass of the disc magnet, the applied axial tensile load *F* is equal to the gravity of disc magnet. Equation (13) is a rough estimation as it assumes a spring with uniform cross-section. In order to verify the fundamental frequency calculated by (13), FEM simulation based on the computed value of the spring is conducted by using COMSOL Multiphysics. The fundamental natural frequency of the EVEH analytically calculated by (13) and simulated by FEM shows good agreement with each other, as shown in Figure 2. The resonant frequency of the spring decreases when the mass of the magnet increases, the decreasing rate is higher when the mass is smaller.

### 2.3. Modal Analysis

**Figure 3:**
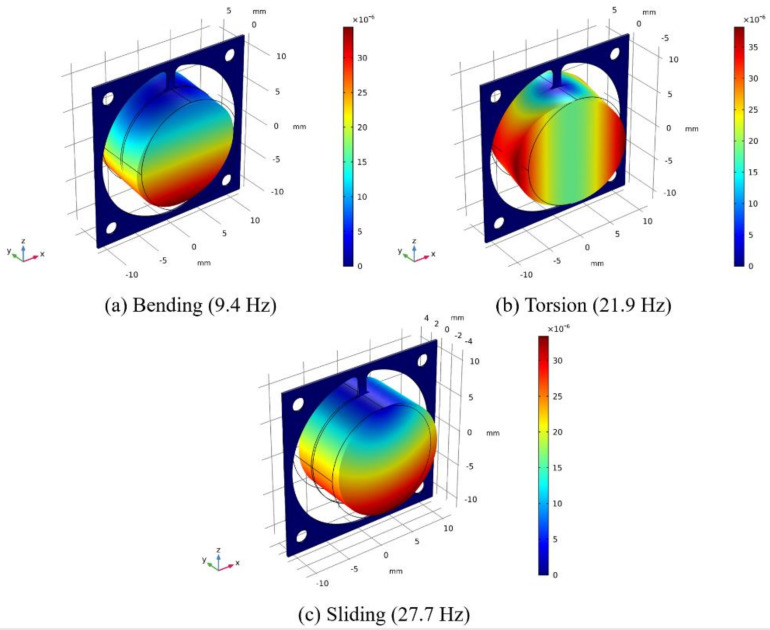
FEM modal analysis of the proposed EVEH.

Due to potential collision between the magnet and the coil, it is difficult to model the behavior of the EVEH device. In order to study the dynamic characteristics of the spring, modal analysis on the resonant mode and frequency of the proposed EVEH is conducted via FEM by using COMSOL Multiphysics, as shown in Figure 3. The model included the gravity of the magnet. Moreover, the primary resonant mode is the bending mode of the spring, similar to the bending of a cantilever. The simulated resonant frequency of the bending mode is 9.4 Hz. Nevertheless, the FEM simulated frequency of the bending mode is not accurate, as the bending mode is strongly influenced by the axial stress in the spring. It is difficult to include the stress in the FEM model since the stress distribution within the spring is rather complex. The main sources of the stress in the spring include mean residual stress and stress gradient from the manufacturing process. The second and the third resonant modes are torsional mode and in-plane sliding mode at 21.9 Hz and 27.7 Hz, respectively. The factors ignored in the FEM mode is the imperfect clamping condition at the spring as well as inaccuracy in the geometry of the device from the assembly process.

## 3. Experimental Procedures

### 3.1. Fabrication and Assembly of the Device

**Figure 4:**
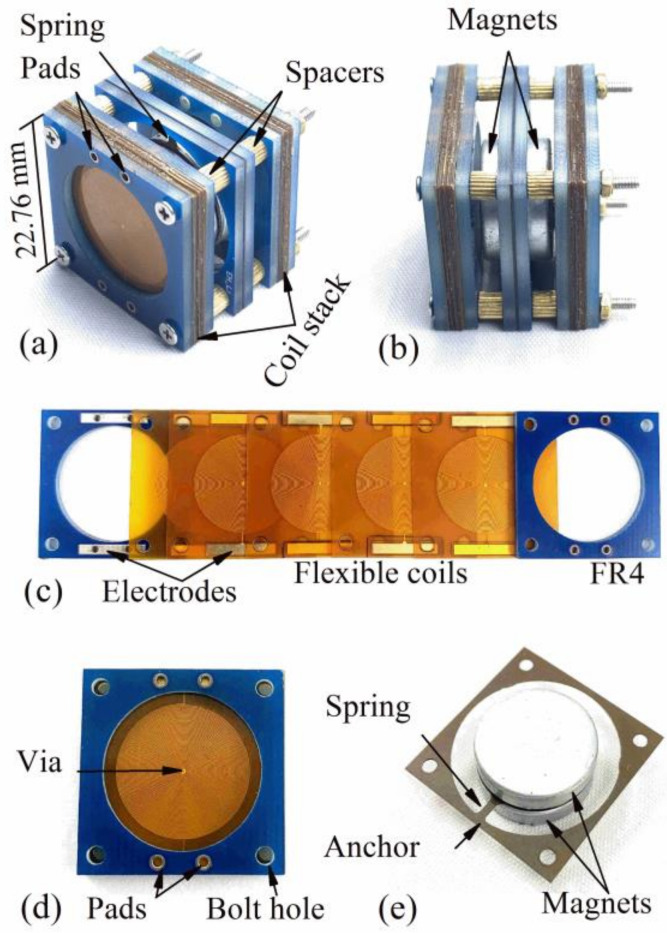
Photos of the (**a**) fabricated EVEH device, (**b**) side view of the EVEH device, (**c**) flexible polymer coils and FR4 boards, (**d**) assembled flexible coil stack, and (**e**) magnets and spring.

The proposed EVEH is successfully fabricated and assembled, as shown in Figure 4. The measured key geometrical parameters of the EVEH are listed in Table 2. Figure 4a shows the assembled EVEH device. Figure 4b shows the side view of the assembled device. The two magnets are suspended between two sets of polymer coil stacks. The magnet layer and coil stacks are assembled by using long bolts and spacers. The distance between the magnets and coils can be adjusted by tuning the thickness of the spacers. Figure 4c,d shows the disassembled and assembled polymer coil stack. In the photos, only 5 coil layers are used for the purpose of simplicity. Spiral copper coils with thickness of 12 μm are electroplated and etched on a 180 μm-thick polyimide substrate to realize the polymer coil layer. Each of the coil layers has two planar coils electroplated on both sides of the polyimide substrates with opposite winding directions, connected by the through-polyimide vias in the center of the coil. Both planar copper coils are covered by thin insulation layer to avoid short circuit when bring into contact. The large electrode pads at the edge of the coil layers connect different layers in series when being stacked. Dummy electrodes with no electrical connection are also electroplated and patterned on the opposite side of the actual electrodes, providing mechanical symmetry and stability in the multi-layer stack. In the EVEH device used for the studies in the next sections, each of the coil stacks consists of 14 coil layers, sandwiched and clamped between two rigid FR4 frames. The FR4 frames are manufactured with standard PCB technologies, with pads for wiring and bolt holes for alignment and clamping. The pads on the FR4 layer are through holes with Sn electroplated on the inner surface and on the back, in order to interface the electrodes on polymer coil and external wiring. Two N52 NdFeB magnets with thicknesses of 4 mm and diameter of 15 mm are fixed together by magnetic force with a polyimide circular plate sandwiched in between. The magnets are suspended by a single string polyimide spring connected to the polyimide circular plate. The polyimide springs and circular plates are realized by laser cutting an entire piece of polyimide foil. The polyimide spring is anchored to a square polyimide frame clamped between two rigid FR4 frames during assembly, as shown in Figure 4e.

### 3.2. Characterization Setup

**Figure 5:**
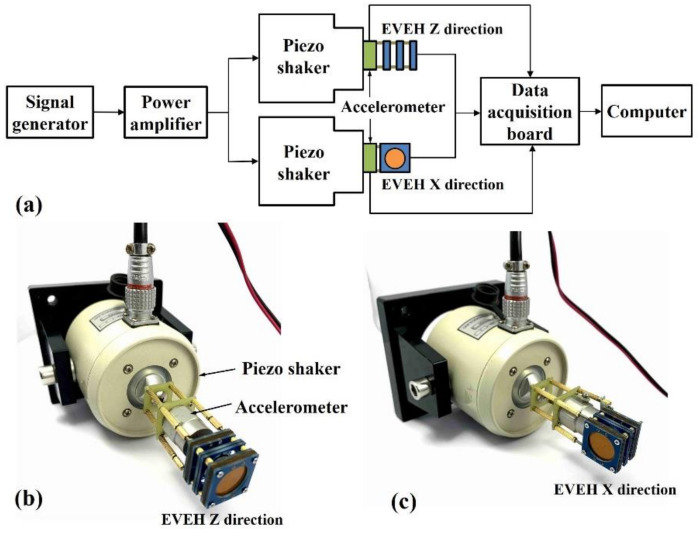
(**a**) Schematic illustration of the setup for characterizing the EVEH device; (**b**) photo of the Z direction measurement setup; and (**c**) photo of the X direction measurement setup.

The fabricated EVEH device is characterized using a setup shown in Figure 5a. The EVEH is mounted on a piezo shaker, which is actuated by a sinusoidal signal from a signal generator and amplified by a power amplifier. An accelerometer is mounted between the EVEH and the shaker to measure the applied acceleration. A NI USB-6211 data acquisition board is used to acquire the output signal of the EVEH, signal generator, power amplifier, and accelerometer with a sampling rate of 10,000 samples per second. A 4th-order Butterworth low-pass filter with a cut-off frequency of 1000 Hz is implemented in the LabVIEW software to remove the high-frequency noise from the signals. When measuring the Z direction operation of the EVEH, the device is mounted in the way that the surface of the disc magnet is perpendicular to the direction of the vibration, as shown in Figure 5b. When measuring the X direction operation of the EVEH, the EVEH is 90 degree rotated so that the surface of the disc magnet aligns with the direction of the vibration, as shown in Figure 5c.

## 4. Results and Discussion

### 4.1. Open-Circuit Characteristics

**Figure 6:**
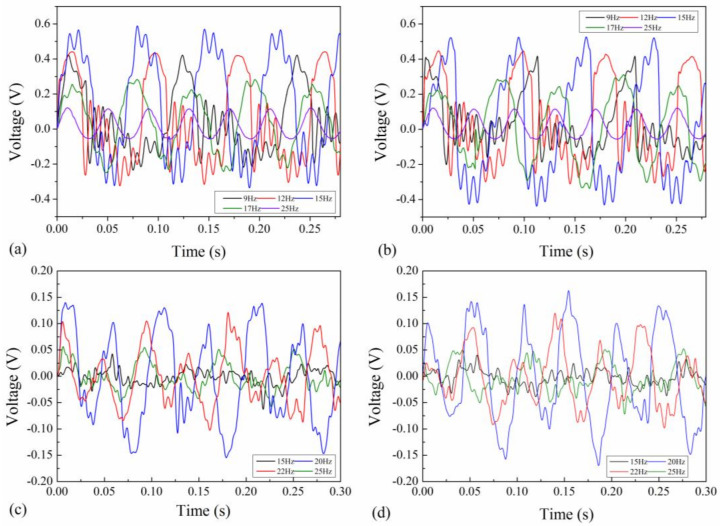
The time-dependent open-circuit output voltage of the EVEH on (**a**) Z-direction in top coil; (**b**) Z-direction in bottom coil; (**c**) X-direction in top coil; and (**d**) X-direction in bottom coil.

The open-circuit output voltage of the EVEH under sinusoidal excitations with amplitude of ±1 g and different frequency is measured as a function of time, as shown in Figure 6. Both the output voltages on Z and X directions show sinusoidal characteristics but with distorted waveforms, i.e., oscillation at the peaks of Z-direction output and varying amplitude of the X-direction output. The oscillation on Z direction is caused by collision between the magnet and the coil. The varying amplitude of the X-direction output is induced by the slight collision between the magnet and FR4 frame. In the next sections, the output voltage values of the EVEH are average values of 10 periods, in order to reduce the inaccuracy from the influence of the oscillations.

The output voltage is small when the frequency of the excitation vibration is far from the resonant frequency of the EVEH. When the vibration frequency gets closer to the resonant frequency of the EVEH, both the output voltage and distortion increase significantly on both Z and X directions, as a result of the increased movement of the magnet. The output voltages of the top and bottom coils are almost identical for the X-direction vibration. In comparison, the Z-direction output voltages of the top and bottom coils have similar positive peaks but slightly different negative peaks, possibly as a result of slightly different movement of the magnet with respect to the top and bottom coils due to collision. At 15 Hz, the maximum output voltages are 0.90 V (top coil) and 0.95 V (bottom coil) on Z direction, 0.28 V (top coil) and 0.31 V (bottom coil) on X direction, respectively. The maximum output voltage on Z direction is larger than on X direction, because the thin and wide spring is easier to be bended (in Z direction) than to be twisted or bended (in X direction). The low-stiffness yet robust polyimide spring enables large and multi degree-of-freedom movement of the magnet while maintains the reliability of the system. Commonly used rigid springs based on silicon and FR4 may not be able to withstand the large movement amplitude of the magnet and mechanical shock induced by the collision between the magnet and coil.

**Figure 7:**
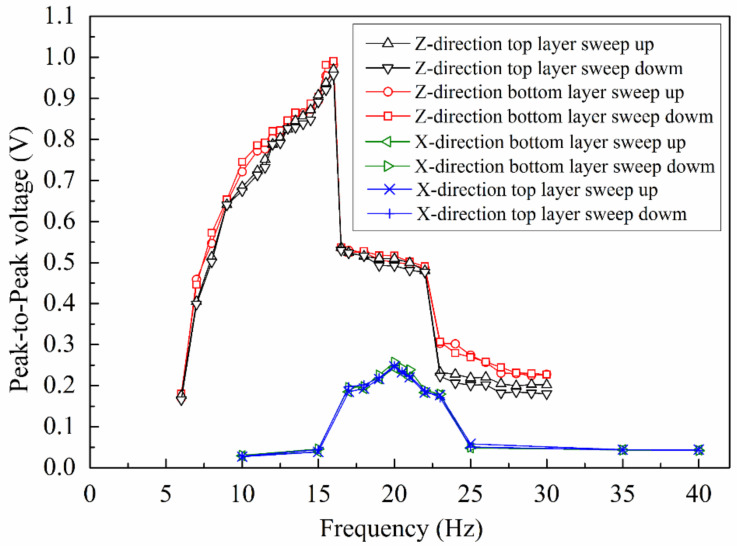
The frequency-dependent open-circuit output voltage of the EVEH in both top and bottom coils, on both Z and X directions.

The peak-to-peak open-circuit output voltage of the EVEH as a function of frequency is shown in Figure 7. The output voltages of the EVEH on Z and X directions are measured by both sweeping up and sweeping down the frequency. The output voltage in both top and bottom coils are measured.

On Z direction, two resonance peaks have been observed at 16 Hz and 20 Hz, respectively. The corresponding resonant modes are bending (16 Hz) and torsional (20 Hz) modes as predicted by the FEM simulation. The resonant frequency of the torsional mode shows good agreement with the FEM simulation (21.9 Hz). However, there is discrepancy between the measured and simulated resonant frequency of the bending mode (16 Hz versus 9.4 Hz), possibly due to the dynamic axial stress in the spring which is not accurately modeled in the FEM. In addition, we have observed the movement of the magnet and discovered that none of the resonant modes of the magnet is a simple motion, as a result of multiple degree of freedom of the spring and the magnet-coil collision. For instance, the bending mode is rather a bending movement combined with torsional movement, and the torsional mode is rather a torsional movement combined with an in-plane sliding motion.

The maximum output voltage of the EVEH is 0.99 V at 16 Hz and 0.52 V at 20 Hz. The difference in the measured maximum outputs in the top and bottom coils is within 0.03 V. There is an abrupt decrease in the output voltage once the frequency is higher than the resonant frequency of the EVEH device. The abrupt decrease often originates from the nonlinear behavior of spring as a result of the axial stress or the overlap between two resonant modes. However, no hysteresis has been observed between the sweep-up and sweep-down (in frequency) characteristics of the EVEH, indicating the absence of nonlinearity in the spring. Therefore, the abrupt decrease may originate from the overlapping between the two resonant modes, which actually effectively expanded the bandwidth of the device. The peak of the torsional mode on Z direction is not obvious, possibly as a result of the overlap between the peak of the torsional mode and the abrupt decrease in amplitude of the bending mode. It is worth noting that the EVEH outputs around 0.2 V of voltage even at the non-resonant frequencies, as a result of the low-stiffness pendulum-like suspension of the magnet. On X direction, only the torsional mode of the EVEH is excited and observed. A maximum output voltage of 0.26 V is observed at the resonant frequency of 20 Hz. The difference in the measured maximum outputs in the top and bottom coils is within 0.01 V. Vibrations on specific directions can only excite specific corresponding resonant modes, e.g., vibration on Z direction is capable of exciting both the bending and torsional mode of the magnet, whereas vibration on X direction is only capable of exciting torsional mode of the magnet. This is determined by the design of the spring: a thin and wide spring is easy to be bended towards the vertical direction rather than the parallel direction of its wide surface. This trend is more obvious when the ratio between the width and thickness is larger.

### 4.2. Impedance Matching

In practical applications, an EVEH device is often used as a power supply and connected to an electric load. Therefore, it is important to study the output characteristics of the EVEH under different load conditions. In this section, the output voltage and power of the EVEH device is measured while its output is connected to load resistors with various values.

**Figure 8:**
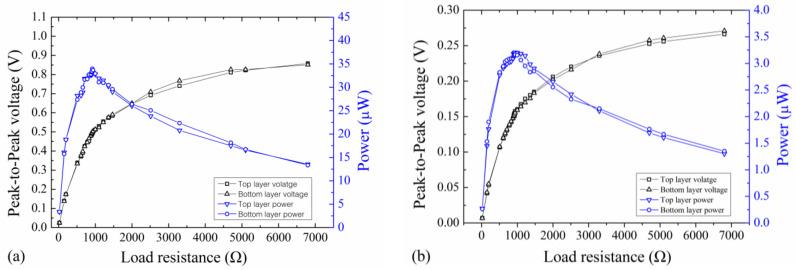
The closed-circuit output voltage and power of the EVEH as a function of load resistance on (**a**) Z direction and (**b**) X direction.

The output peak-to-peak closed-circuit voltage and power of the EVEH device on both Z and X directions is plotted versus the load resistance, as shown in Figure 8. The data in the figure are obtained with an applied excitation vibration of ±1 g and frequencies of 16 Hz (Z direction) and 20 Hz (X direction). On Z direction, the maximum output power of the EVEH is 33.7 μW with an optimal load resistance of 940 Ω. On X direction, the maximum output power of the EVEH is 3.2 μW with an optimal load resistance of 940 Ω. The power on X direction is 10.5 times smaller than on Z direction, because the power is proportional to the square of the voltage. The optimal load value is similar as the measured coil resistance of 937 Ω. The slight difference between the optimal load and the coils resistance originates from the measurement inaccuracy as well as the ignored resistance of the wires.

### 4.3. Closed-Circuit Characteristics

**Figure 9:**
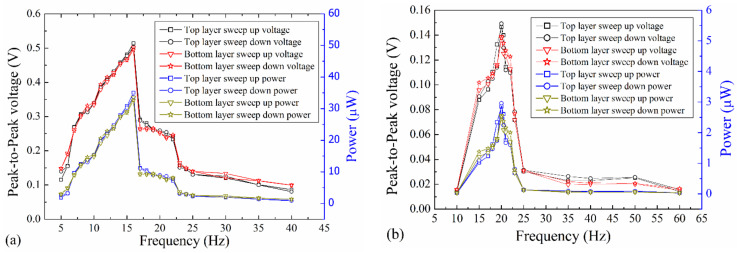
The closed-circuit output voltage of the EVEH as a function of frequency on (**a**) Z direction and (**b**) X direction.

The closed-circuit output voltage and power of the EVEH as a function of frequency is shown in Figure 9. Figure 9a shows the output voltage and power on Z direction under an applied vibration excitation of ±1 g. Two resonance modes of bending and torsion are visible in the figure. At the dominant resonant frequency of 16 Hz, a maximum output voltage of 0.51 V and a power of 35.1 μW are measured in the top coil. Correspondingly, a maximum output voltage of 0.50 V and a power of 33.6 μW are measured in the bottom coil at 16 Hz. Combining output of the top and bottom coils, the device is capable of generating a power of 68.7 μW in total. No hysteresis has been observed in the frequency sweep-up and sweep-down measurements. The maximum output power of 68.7 μW is 429 times higher than the maximum output power of 160 nW at 1 g vibration of the EVEH with similar technology reported in 2016 [8] and 159 times higher than the maximum output power of 0.43 μW of the EVEH with similar technology and size reported in 2017 [6]. The output power is lower than the work based on stacked coils reported in 2018 [7], due to the increased magnet-coil distance. Nevertheless, the EVEH presented in this work is capable of harvesting vibrations on two different directions, which has not been realized in previous works. With a volume of 8.1 cm^3^, the proposed device has a normalized power density (NPD) of 8.48 μW/cm^3^·g^2^, which is higher than several similar polymer-based energy harvesters, e.g., 42 times higher than the NPD of 0.2 μW/cm^3^·g^2^ of an electromagnetic/triboelectric hybrid polymer energy harvester [20], 1696 times higher than the NPD of 0.005 μW/cm^3^·g^2^ of an electromagnetic energy harvester based on flexible coil and liquid spring [21]. In [22], the NPD of an electromagnetic energy harvester based on magnetic spring (1.97 mW/cm^3^·g^2^) is 232 times higher than this work, because it works at very low acceleration. The magnetic spring of the device enables operation at very low acceleration (0.4 g) but increases the volume of the device (194.5 cm^3^, 24 times larger than the proposed EVEH device). Nevertheless, there are other advantages of the magnetic spring technology, e.g., extended lifetime and reduced negative stiffness spring [23]. The combination of non-linear resonance and multi-mode operation of the EVEH on Z direction provide a higher bandwidth than traditional EVEH devices with single resonance mode. Figure 9b shows the output voltage and power on X direction under an applied vibration excitation of ±1.5 g. On X direction, the dominant mode is the torsional mode at 20 Hz, where a maximum output voltage of 0.15 V and power of 2.96 μW are measured in the top coil and a maximum output voltage of 0.14 V and power of 2.56 μW are measured in the bottom coil. The bending mode has a very small amplitude, evidenced by the slight change in the slope of the curve around 16 Hz. The resonant frequency of the proposed device is among the lowest of the reported electromagnetic energy harvesters. If further reduction in the operation frequency is needed, one may consider the use of non-resonant energy harvesters such as the triboelectric-based devices [23,24].

### 4.4. Acceleration Test

**Figure 10:**
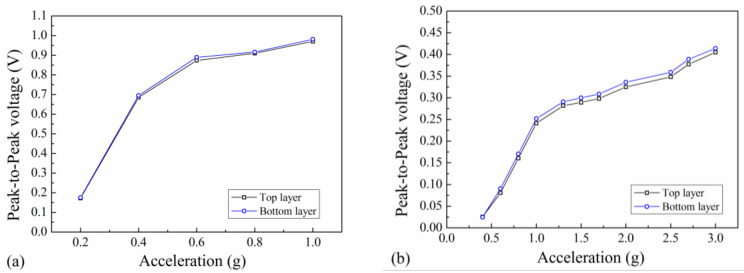
The open-circuit output voltage of the EVEH as a function of acceleration level on (**a**) Z-direction and (**b**) X-direction.

The output of the EVEH under different vibration levels is studied on both Z and X directions. On Z direction, the open-circuit output voltage of the EVEH is measured under excitation of ±0.2–±1 g, as shown in Figure 10a. The output voltage increases as the vibration amplitude increases. The voltage increases first rapidly between ±0.2 g and ±0.4 g and then gradually between ±0.4 g and ±1 g. The saturation in the increase of output voltage is due to the limited motion of the magnet that can be achieved within the small coil-magnet gap. The output voltage increases from 0.18 V at ±0.2 g to 0.98 V at ±1 g. On X direction, the open-circuit output voltage of the EVEH is measured under excitation of ±0.4–±3 g, as shown in Figure 10b. The output voltage increases rapidly in the range of ±0.4–±1 g, and saturates gradually after ±1 g. The output voltage increases from 0.02 V at ±0.4 g to 0.41 V at ±3 g. The dependence of the output on acceleration suggests that the EVEH is suitable to be used at low acceleration level on Z direction and at high acceleration on X direction. Using the EVEH at high acceleration level may induce intensive collision between the magnet and the coil, which will deteriorate the reliability of the device.

## 5. Conclusions

In this work, a low frequency electromagnetic vibrational energy harvester (EVEH) with two degrees of freedom and two resonant modes is designed, fabricated, and characterized. The proposed EVEH is implemented by suspending a disc magnet in a pendulum fashion by a polymer spring between two sets of polymer coil stacks. The fabricated EVEH is capable of harvesting vibration energy on two directions with an extended bandwidth. With a sinusoidal acceleration of ±1 g in amplitude on Z direction, a peak-to-peak closed-circuit output voltage of 0.51 V (open-circuit voltage: 1 V) and an output power of 35.1 μW are measured, at the resonant frequency of 16 Hz. The two coils in the device output a power of 68.7 μW in total. With a sinusoidal acceleration of ±1.5 g in amplitude on X direction, a peak-to-peak output voltage of 0.14 V and power of 2.56 μW are measured, at the resonant frequency of 20 Hz. In future work, the size and structure of the proposed EVEH can be still optimized from the aspect of design and fabrication process. In addition, despite the excellent energy harvesting capabilities, the proposed EVEH does have issues such as magnet-coil collision, which might reduce the reliability and lifetime of the EVEH device. These issues must be considered in the future design of the EVEH devices.

## Figures and Tables

**Table 1:** Critical parameters used the resonant frequency calculation.

Parameters	Values
*k_eff_* (N∙m)	25.2
*P_cr_* (N)	2.514
*m* (g)	0.0754

**Table 2:** Measured geometrical parameters of the fabricated EVEH.

Parameters	Measured Values
Size of the device (L × W × H) (mm)	22.8 × 22.8 × 15.6
Coil thickness (mm)	0.012
Coil line width (mm)	0.05
Spacing between coil lines (mm)	0.05
Number of coil turns per layer	98
Length of the spring (mm)	2.8
Width of the spring (mm)	1.0
Thickness of the spring (mm)	0.3
Diameter of the magnet (mm)	15.00
Thickness of the magnet (mm)	4
Magnet-coil gap (mm)	2.61
